# Bioactivity Profiles on 15 Different Effect Mechanisms for 15 Golden Root Products via High-Performance Thin-Layer Chromatography, Planar Assays, and High-Resolution Mass Spectrometry

**DOI:** 10.3390/molecules28041535

**Published:** 2023-02-05

**Authors:** Hanna Nikolaichuk, Irena M. Choma, Gertrud E. Morlock

**Affiliations:** 1 Chair of Food Science, Institute of Nutritional Science, Justus Liebig University Giessen, Heinrich-Buff-Ring 26-32, 35392 Giessen, Germany; 2 Department of Chromatography, Faculty of Chemistry, Maria Curie-Sklodowska University, Maria Curie-Sklodowska Sq. 3, 20031 Lublin, Poland; 3 Department of Bioanalytics, Faculty of Biomedicine, Medical University of Lublin, Jaczewskiego St. 8b, 20090 Lublin, Poland

**Keywords:** *Rhodiola rosea*, antibacterial, antimicrobial, enzyme inhibitor, antioxidant, genotoxin, estrogen, androgen, agonist, antagonist, endocrine activity, bioassay, biochemical assay, effect-directed analysis, HPTLC-EDA, HPTLC−HPLC−HESI-HRMS

## Abstract

Planar chromatography has recently been combined with six different effect-directed assays for three golden root (*Rhodiola rosea* L.) samples. However, the profiles obtained showed an intense tailing, making zone differentiation impossible. The profiling was therefore improved to allow for the detection of individual bioactive compounds, and the range of samples was extended to 15 commercial golden root products. Further effect-directed assays were studied providing information on 15 different effect mechanisms, i.e., (1) tyrosinase, (2) acetylcholinesterase, (3) butyrylcholinesterase, (4) β-glucuronidase, and (5) α-amylase inhibition, as well as endocrine activity via the triplex planar yeast antagonist-verified (6–8) estrogen or (9–11) androgen screen, (12) genotoxicity via the planar SOS-Umu-C bioassay, antimicrobial activity against (13) Gram-negative *Aliivibrio fischeri* and (14) Gram-positive *Bacillus subtilis* bacteria, and (15) antioxidative activity (DPPH• radical scavengers). Most of the golden root profiles obtained were characteristic, but some samples differed substantially. The United States Pharmacopeia reference product showed medium activity in most of the assays. The six most active compound zones were further characterized using high-resolution mass spectrometry, and the mass signals obtained were tentatively assigned to molecular formulae. In addition to confirming the known activities, this study is the first to report that golden root constituents inhibit butyrylcholinesterase (rosin was tentatively assigned), β-glucuronidase (rosavin, rosarin, rosiridin, viridoside, and salidroside were tentatively assigned), and α-amylase (stearic acid and palmitic acid were tentatively assigned) and that they are genotoxic (hydroquinone was tentatively assigned) and are both agonistic and antagonistic endocrine active.

## 1. Introduction

Golden root, botanically known as *Rhodiola rosea* L., is a herbal remedy commonly used in traditional Chinese medicine and in European and Asiatic healing systems [1]. It is a perennial flowering plant from the Crassulaceae family. Its extracts are well-known as natural adaptogens, which increase adaptability, resilience, and organisms’ ability to survive stress [2,3,4]. Recent in vitro and in vivo studies indicated that *R. rosea* extracts exhibit a wide variety of medicinal properties and biological activities. These include anti-aging [5], anti-inflammatory [6], anti-stress [7], anti-depression [8], antioxidant [9,10], anti-fatigue [11], anti-viral [12], and anti-osteoporosis [13] properties, as well as effects that promote increased immunity [14,15,16,17]. The ergogenic properties of golden root extracts include the improvement of mental and physical conditions, memory, mood, energy metabolism, and cognitive function [18,19,20,21,22]. Further studies reported a possible role for *R. rosea* extracts in the treatment of cardiovascular [23] and neurodegenerative diseases [24,25,26,27,28], type 2 diabetes [29,30], obesity [31], and cancer [32,33]. Most of these properties are related to salidroside, one of the known major bioactive components in golden root.

Furthermore, *R. rosea* extract was highlighted as a natural selective estrogen receptor modulator beneficial in treating and preventing menopause and related symptoms such as fatigue, stress, depression, osteoporosis, and cancer. In comparison with its synthetic counterparts, *R. rosea* possesses fewer side effects [34]. Another study indicated that constituents of golden root such as gossypetin, herbacetin, and (+)-lariciresinol docked strongly to both of the two estrogen receptors ERα and ERβ [35]. However, further studies are needed, as reports of the estrogenic properties of *R. rosea* are not consistent, and the differences are probably related to different plant sources, extraction protocols, and modes of administration. Moreover, golden root polysaccharides are reported to have protective effects on boar sperm; these include improved motility, mitochondrial activity, acrosomal integrity, and plasma membrane integrity [36]. Though its protective mechanisms remain unclear, *R. rosea* extract could be a potential cryoprotectant in freezing semen. At very high minimum inhibitory concentration values ranging from 1 to 32 mg/mL determined by using the serial microdilution method, *R. rosea* extract inhibited *Staphylococcus epidermidis*, *Staphylococcus aureus*, *Klebsiella pneumoniae*, *Bacillus cereus*, *Bacillus subtilis*, *Listeria monocytogenes*, *Enterobacter aerogenes*, *Escherichia coli*, *Proteus mirabilis*, and *Pseudomonas aeruginosa*. The results indicated that strains of Gram-positive bacteria were more sensitive to the preparations of golden root than Gram-negative bacteria, a finding attributed to the presence of essential oils [37]. In another study of *R. rosea* [38], gossypetin-7-*O*-L-rhamnopyranoside and rhodioflavonoside, which are antibacterial against *Staphylococcus aureus*, were detected at minimum inhibitory concentrations of 50 µg/mL and 100 µg/mL, respectively. However, there is a lack of information on the compounds responsible, as the antibacterial activity of *R. rosea* is rarely studied.

Thin-layer chromatography−effect-directed analysis (TLC−EDA) via six different assays (i.e., acetylcholinesterase, lipase, α-glucosidase, and tyrosinase inhibition assays in addition to antibacterial and antioxidant assays) had already been used for authenticity and bioactivity screening of the golden root samples and the marker compounds rosavin, salidroside, and *p*-tyrosol [39]. However, the effect profiles obtained were unsatisfactory, since no compound differentiation was possible, owing to a highly intense zone tailing. Nevertheless, seven fraction areas were scraped off, eluted, and analyzed offline using high-performance liquid chromatography−electrospray ionization mass spectrometry (HPLC−ESI-MS) to match them with known compounds. The co-applied marker rosavin showed activity against α-glucosidase, tyrosinase, and *Bacillus subtilis*. Salidroside and *p*-tyrosol were proven to be antioxidants, as well as α-glucosidase inhibitors. Salidroside also exhibited antibacterial activity. However, the dominant effect responses in the sample zone tailing could not be explained by the three reference compounds; this suggested that other compounds were responsible for the main bioactivity.

This study aimed to improve the profiling and to develop a non-target effect-directed screening that is able to separate and thus visualize the individual bioactive compounds in the complex golden root samples. The profiling was extended to 15 golden root samples available on the market, including a United States Pharmacopeia (USP) reference product, in order to learn more about product variances and differences in the bioactivity profiles. The samples were investigated using high-performance thin-layer chromatography (HPTLC) combined with 11 different assays (among these were 2 triplex bioassays), indicating 15 effect mechanisms. The six most bioactive zones were further characterized via a straightforward online hyphenation, i.e., via heart-cut zone elution, a desalting loop (freed from assay salts), and orthogonal HPLC separation to heated electrospray ionization high-resolution mass spectrometry (HPTLC−HPLC−HESI-HRMS).

## 2. Results and Discussion

### 2.1. Optimization of the Effect-Directed Profiling Method

To widen the sample range, 15 golden root samples were bought from different Polish and German vendors (Table 1). Thus, information was obtained on the current product variants on the market and the differences in their bioactivity profiles. Similarly to the previous study [39], the samples were extracted with methanol−water 4:1 (*v*/*v*), and 4 µL of each extract was applied (400 µg/band). The non-target effect-directed profiling was developed with no standard or marker compounds in mind. The Gram-negative *Aliivibrio fischeri* bioassay, which, based on our experience in other studies, detects a high number of bioactive zones by reducing bioluminescence in real time, was used to evaluate the 20 mobile phases studied with regard to bioactive compound separation (Supplementary Material Table S1). The mobile phase ethyl acetate–methanol–water 77:13:10 [39] was used as a basis for optimization. The zone tailing was substantially reduced by adding acetic acid to the mobile phase system. However, this required an additional neutralization step prior to the assay application, since most assays do not tolerate acidic traces that remain adsorbed (after plate drying). The zones were comparatively sharper, and when the proportion of water in the mobile phase system was increased, the polar compounds migrated out of the start zone, as is evident in the HPTLC chromatogram at UV 254 nm (Supplementary Material Table S1). Based on the *A. fischeri* bioautogram, the mobile phase consisting of ethyl acetate–methanol–water–acetic acid 70:15:15:1 was found to be suitable in initiating the intended effect-directed profiling. After the application and separation of all 15 products (Table 1), plate neutralization was performed in order to neutralize any remaining traces of acidic solvent. 

The neutralized chromatogram was prepared 11 times (with adjustments for specific assays as described), as 11 effect-directed assays were applied, indicating 15 different response mechanisms due to 2 triplex bioassays. Considering the medicinal properties previously mentioned, the activity of the 15 golden root samples was evaluated with regard to their effects as antibacterials against Gram-positive *B. subtilis* and Gram-negative *A. fischeri*, and inhibitors of tyrosinase, acetylcholinesterase, butyrylcholinesterase, β-glucuronidase, and α-amylase, as well as antioxidants detected via the 2,2–diphenyl–1–picrylhydrazyl radical (DPPH•) scavenging assay. In particular, information was sought on the presence of genotoxic compounds in the golden root samples via the SOS-Umu-C bioassay and agonistic and antagonistic endocrine compounds via the planar triplex yeast antagonist-verified estrogen/androgen screen (pYAVES/pYAVAS) bioassay.

### 2.2. Effect-Directed Profiling of 15 Golden Root Samples

#### 2.2.1. *Aliivibrio fischeri* Bioassay

**Figure 1:**
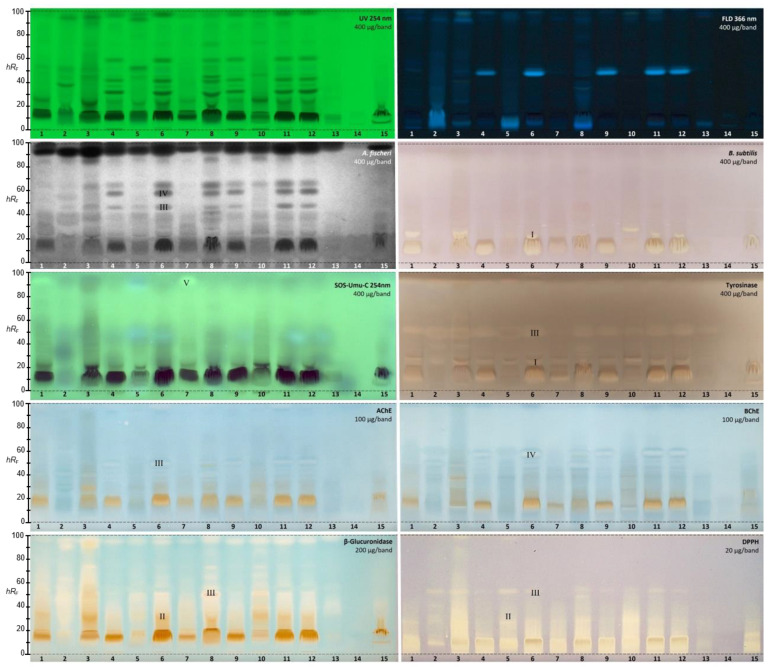
HPTLC chromatograms at UV 254 nm and FLD 366 nm and (bio)autograms of 15 golden root product extracts (Table 1, IDs 1−15, 20−400 µg/band depending on the assay as indicated) separated on HPTLC plates silica gel 60 F_254_ (without F_254_ for the SOS-Umu-C bioassay) using ethyl acetate–methanol–water–acetic acid 70:15:15:1 (*v*/*v*/*v*/*v*) and detected after the respective assay application via the instant bioluminescence (*A. fischeri*) at FLD 254 nm (SOS-Umu-C bioassay) or white light illumination; zones marked (I−V) were subjected to HPTLC−HPLC−HESI-HRMS recording.

The 15 golden root extracts (400 µg/band each) revealed antibacterial activity against Gram-negative *A. fischeri* in the bioautogram (Figure 1). Up to five different prominent antibacterial (dark) zones were observed on the bioluminescent plate background, where the intrinsic (instant) green-blue bioluminescence was depicted as a grayscale image. The different products showed clear differences in the antibacterial profiles; e.g., ID 14 did not provide a response at all, and IDs 2, 5, 7, 13, and 15 reacted with a much weaker antibacterial effect in comparison with the other products. The USP reference standard of *Rhodiola rosea* L. root and rhizome (ID 8) showed antibacterial effects comparable to product IDs 6, 11, and 12.

#### 2.2.2. *Bacillus subtilis* Bioassay

The incubation time for the *B. subtilis* bioassay was reduced by 15 h, from the 17.5 h used previously [39] to 2.5 h [40]. The overall antibacterial response against Gram-positive *B. subtilis*, observed in the bioautogram under white light illumination as colorless (white) zones against a purple plate background, was comparatively weaker than that against *A. fischeri*. The main response was evident in the polar compound range and was similar to the previous dark zones active against *A. fischeri*. Again, the observed antibacterial profiles differed clearly between the products. The USP reference product ID 8 showed only a weak zone at *hR_F_* 10, which was not as intense as in IDs 1, 3, 4, 6, 9, 11, and 12. Some products (IDs 2, 5, 13, and 14) were not active at all at the given amounts.

#### 2.2.3. SOS-Umu-C Genotoxicity Bioassay

The planar SOS-Umu-C bioautogram indicated a genotoxic compound at *hR_F_* 93 as a bright green fluorescent zone against a less green fluorescent plate background in two samples (Figure 1, IDs 1 and 7). The genotoxic zone was clearly detectable despite the diffuse zones in the bioautogram caused by the 3.5 h long incubation. Later, this genotoxic compound zone was identified using HPTLC−HPLC−HESI-HRMS. Fluorescein-di-β-D-galactopyranoside (FDG) was chosen as a substrate for the glucosidase released upon contact with the genetically modified *Salmonella typhimiurium* strain with a genotoxin, since it provided the fluorescein, which is the green fluorescent end product. This was advantageous owing to the given natively blue fluorescent compounds in the separated golden root samples (HPTLC chromatogram at FLD 366 nm), which are able to shine through in the bioautogram (Figure 1). The formed fluorescein was detected at FLD 254 nm; this required the use of HPTLC plates silica gel 60 without the fluorescence indicator F_254_, in order to avoid any measurement-signal interference. The presence of genotoxic compounds in two of the golden root supplement products on the market highlighted the importance of the effect-directed profiling as a quality control measure for plant-based supplements before they are made available to consumers. Further research is needed to clarify whether the genotoxic response is also detected in other batches. Luckily, the USP reference product ID 8 did not contain a genotoxin.

#### 2.2.4. Tyrosinase Inhibition Assay

The tyrosinase inhibition autogram of the 15 golden root products (Figure 1) revealed (as colorless inhibiting zones on a grey plate background) several more polar inhibition zones at *hR_F_* 10 and 20, and further such zones at *hR_F_* 51 and 90. Again, in contrast to most samples with up to three intense responses, product ID 14 showed no activity at all, and product IDs 13 and 15 only a very weak inhibition. The USP reference product ID 8 revealed all the above-mentioned inhibition zones (*hR_F_* 10, 20, 51, and 90). The inhibiting compound zones at *hR_F_* 20 and 51 were subjected to HPTLC−HPLC−HESI-HRMS.

#### 2.2.5. Acetyl- and Butyrylcholinesterase Inhibition Assays

The acetylcholinesterase (AChE) and butyrylcholinesterase (BChE) inhibition assays were often applied using Fast Blue B salt as a chromogenic reagent for the enzyme−substrate reaction. However, the sample matrix can react with polyphenols and generate an interfering brown color. A test of the reaction between golden root extract and Fast Blue B salt confirmed our hypothesis (Supplementary Material Figure S1). Thus, in contrast to [39], the AChE and BChE inhibition assays were performed using indoxyl acetate as the substrate, resulting in colorless inhibiting zones against an indigo blue plate background (Figure 1). The amount of golden root applied was reduced by a factor of 4 from 400 to 100 µg/band to avoid signal overload. In half of all the samples, up to two clear inhibitors were observed: the lower zone at *hR_F_* 51 was an AChE inhibitor, as evident in the AChE autogram, in contrast to the upper zone at *hR_F_* 61, which preferably inhibited the BChE, as observed in the respective BChE autogram. The USP reference product ID 8 showed inhibition of the AChE at *hR_F_* 40 and 51 and of BChE at *hR_F_* 51 and 61. Again, the same products (IDs 13−15) showed either no inhibition potential or the weakest inhibition potential among all the samples studied. The two inhibiting compound zones at *hR_F_* 51 and 61 were subjected to subsequent HPTLC−HPLC−HESI-HRMS analysis.

#### 2.2.6. β-Glucuronidase Inhibition Assay

For the β-glucuronidase inhibition assay (Figure 1), the applied amount of golden root was reduced to 200 µg/band in each case (to avoid signal overload). In the autogram, β-glucuronidase inhibitors were revealed as colorless inhibiting zones against an indigo blue plate background when 5-bromo-4-chloro-3-indonyl-β-D-glucuronide was used as the substrate for the enzyme. Diffuse tailing inhibition zones were detected. The 1% acetic acid content of the mobile phase was too low to sharpen the very polar active compound zones. In a future study, these will be able to be focused by means of using a stronger acid and a higher acid proportion in the mobile phase (e.g., using 10% formic acid). Since this would alter the separation and profile, we tolerated the zone tailing for reasons of comparison. Nevertheless, clear inhibition differences were evident between the products, and one half of the products exhibited greater potency than the other half. The USP reference product ID 8 was medium in activity compared with all other samples. Again, the same products (IDs 13−15) showed either no β-glucuronidase inhibition potential or the weakest β-glucuronidase inhibition potential.

#### 2.2.7. DPPH• Scavenging Assay

The DPPH• autogram (Figure 1) showed that golden root extracts possessed high radical scavenging, and thus antioxidant, activity, evident as yellow zones on a purple plate background. For an initial extract amount of 400 µg/band, the autogram was totally overloaded (Supplementary Material Figure S2). Consequently, the applied extract amount was reduced by a factor of 20, and even with only 20 µg/band applied, many tracks still appeared overloaded. Similarly to the β-glucuronidase inhibition assay, tailing antioxidative zones were detected, but, based on our experience in other projects, these could be sharpened in future using 10% formic acid instead of 1% acetic acid. Again, the USP reference product ID 8 was medium in activity compared with all other samples. Product ID 14 showed no antioxidant activity even at 400 µg/band (Supplementary Material Figure S2). Product ID 13 showed almost no activity, and ID 2 was third weakest product in antioxidant activity.

#### 2.2.8. α-Glucosidase and β-Glucosidase Inhibition Assays

The α/β-glucosidase inhibition activity was studied using 4-methylumbelliferyl-α/β-D-glucopyranoside as the substrate. However, previous results [41] had already revealed that substrate detection was not functioning correctly, owing to the direct reaction of sample compounds with the substrate. Here, this assumed false positive reaction was studied in detail and proven by performing the detection with only the substrate present, and with no enzyme (Supplementary Material Figure S3). Consequently, this assay detection still requires further optimization for its application to golden root samples.

#### 2.2.9. α-Amylase Inhibition Assay

**Figure 2:**
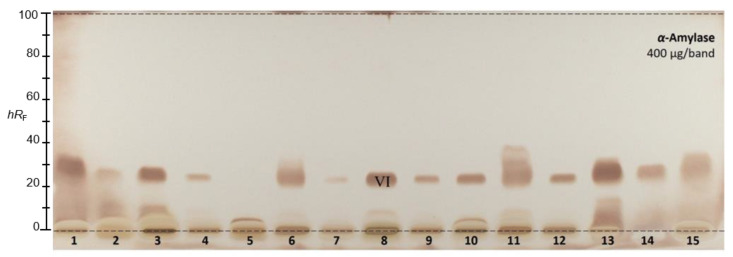
HPTLC–α-amylase inhibition autogram of 15 golden root product extracts (400 µg/band each) separated on HPTLC plates silica gel 60 F_254_ using ethyl acetate–*n*-hexane 3:7 (*v*/*v*) and detected at white light illumination after the assay application; the zone marked (VI) was subjected to HPTLC−HPLC−HESI-HRMS recording.

A different mobile phase was required for the α-amylase inhibition assay (Figure 2), since a pretest showed that all the sample responses (observed as brown zones on a bright plate background) were present in the solvent front (Supplementary Material Figure S4). The elution power was too strong for the α-amylase inhibiting compounds, and it was thus reduced in order to obtain ethyl acetate–*n*-hexane 3:7 (*v*/*v*). All 15 golden root products revealed α-amylase inhibition activity. In the autogram, two active zones were detected: one at the start zone and another prominent one at *hR_F_* 23. The latter compound zone (*hR_F_* 23) was subjected to HPTLC−HPLC−HESI-HRMS recording.

#### 2.2.10. pYAVAS and pYAVES Bioassays

**Figure 3:**
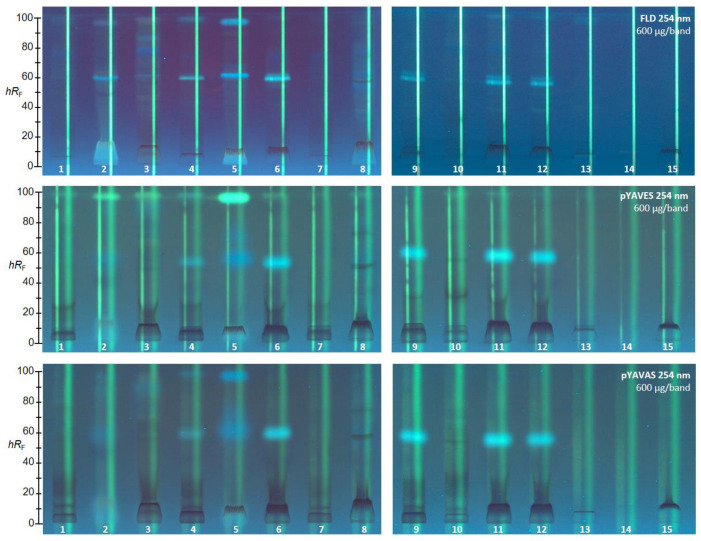
HPTLC chromatograms and HPTLC−pYAVES/pYAVAS−FLD triplex bioautograms showing agonistic, antagonistic, and false-positive antagonistic endocrine effects in 15 golden root extracts (12 mm band, 6 µL/band, 600 µg/band each) separated on HPTLC plates silica gel 60 using ethyl acetate–methanol–water 70:15:15 (*v*/*v*/*v*) and detected at FLD 254 nm before (for comparison) and after the respective bioassay.

Possible endocrine effects in the 15 golden root products were studied in the triplex agonistic/antagonistic pYAVES/pYAVAS bioassays (Figure 3) [42]. Acetic acid was eliminated from the mobile phase to simplify the protocol for the following triplex bioassays. In respect of zone fixation, two additional layer treatments were newly included to avoid diffusion during the 3 or 4 h long incubation. Thus, the two stripes, which were applied along each separated sample track before the bioassay application, remained sharp, which was helpful for the evaluation of the agonistic/antagonistic response profiles in the triplex bioautogram [42]. As for the SOS-Umu-C genotoxicity bioassay, FDG was chosen as a substrate for the released glucosidase (upon contact of the respective genetically modified *Saccharomyces cerevisiae* strain with an agonist), since it advantageously generated the green fluorescent fluorescein end product as previously mentioned. Consequently, plates without F_254_ had to be used here as well. The 15 products were applied at higher amounts (600 µg/band each) owing to the longer application band (12 mm band) required to provide sufficient space for the application of the two stripes. To provide the detection of both the estrogenic and the antiestrogenic activity on the same plate via the triplex pYAVES bioassay, two stripes were applied along each separated sample track before the bioassay application. The first agonist stripe was 17β-estradiol (detectable first after the bioassay), and the second end-product stripe was fluorescein (directly detectable; see Figure 3).

After the bioassay application, a clear estrogenic compound zone was detected as a green fluorescent zone at *hR_F_* 99 close to the solvent front. In particular, in product ID 5 the response was strongest, followed by product ID 2, then ID 3. However, further products (IDs 1, 4, 6, 8, 10 and 11) also showed a weak response. A verified antiestrogenic activity, evident as a reduction in fluorescence on the first 17β-estradiol stripe (biologically-induced reduction), was observed in many samples, for example, in product IDs 1, 4, 6, and 7 at *hR_F_* 90. Product ID 9 showed several (and comparatively the most) antiestrogenic compounds. These antagonistic effects were unequivocally verified by the co-applied second fluorescein stripe, which was not reduced in the fluorescence. In case of false positive responses (caused physico-chemically), the second fluorescein stripe would be reduced in fluorescence. In order to evaluate androgenic and antiandrogenic activity, the triplex pYAVAS bioassay was used analogously. However, the first agonist stripe was the testosterone. As a result, no androgens or antiandrogens were revealed, even for the applied 600 µg/band golden root extract (Figure 3).

### 2.3. Characterization of Six Active Compound Zones Using HPTLC−HPLC−HESI-HRMS

The six most active compound zones (**I**–**V** in Figure 1 according to ascending *hR_F_*, as well as **VI** in Figure 2 with a mobile phase of reduced solvent strength) were selected and subjected to HPTLC−HPLC−HESI-HRMS recording [40,42,43]. Based on data from the literature and the HRMS spectra obtained in the negative and positive ionization modes, molecular formulae were tentatively assigned (Table 2, Supplementary Material Figure S5).

The first active zone at *hR_F_* 20 with antibacterial activity against *B. subtilis* and inhibition of tyrosinase was tentatively assigned to rhodioloside D, since the recorded mass signals at *m*/*z* 409.2084 [M+CH_3_COO]^−^ and *m*/*z* 373.1828 [M+Na]^+^ matched this marker compound. The second active zone at *hR_F_ 35* with strong antioxidant and β-glucuronidase inhibition activity revealed two mass signals at *m*/*z* 427.1610 [M-H]^−^ and *m*/*z* 451.1576 [M+Na]^+^, tentatively assigned to the two marker compounds, rosavin and rosarin. The third active zone at *hR_F_* 51 with antioxidant, antibacterial (against *A. fischeri*), and AChE, tyrosinase, and β-glucuronidase-inhibiting activity indicated three constituents of golden root, i.e., rosiridin with mass signals at *m/z* 391.1978 [M+CH_3_COO]^−^ and *m*/*z* 355.1722 [M+Na]^+^, viridoside at *m*/*z* 359.1351 [M+CHOO]^−^ and *m*/*z* 337.1258 [M+Na]^+^, and salidroside at *m*/*z* 299.1139 [M-H]^−^ and 323.1099 [M+Na]^+^. The fourth active zone at *hR_F_* 61 inhibiting BChE and active against *A. fischeri* was tentatively assigned to rosin with mass signals at *m*/*z* 355.1399 [M+CH_3_COO]^−^ and *m*/*z* 319.1146 [M+Na]^+^. All the compounds mentioned were responsible for the activity and used for the standardization of golden root raw material [1,2]. The mass spectra from the fifth genotoxic zone at *hR_F_* 93 exhibited two mass signals at *m*/*z* 109.0294 [M-H]^−^ and 111.0444 [M+H]^+^. Thus, hydroquinone was tentatively assigned as the compound responsible for the genotoxic activity in the golden root of which the genotoxic potential is known [44]. The mass spectra in the negative and positive ionization modes for the sixth α-amylase inhibiting compound zone at *hR_F_* 23 (via mobile phase reduced in elution strength) exhibited signals corresponding to stearic acid and palmitic acid. These compounds were tentatively assigned as prominent α-amylase inhibitors in almost all the golden root products.

Rosavin, rosarin, rosin, salidroside, viridoside, and rosiridin are the major constituents of golden root with important activities. In particular, rosavin and salidroside have frequently been used for the standardization of golden root products, as well as for quality evaluation and the detection of possible adulteration. The standard concentration ratio of rosavin to salidroside is 3 to 1. The content of these compounds in golden root and rhizome extracts depends on several factors. However, the factors that have the most impact on the content of these compounds are harvest time, long-term vegetative propagation, and genetic diversity [39,45,46,47].

## 3. Materials and Methods

### 3.1. Chemicals

Acetic acid, bovine serum albumin, caffeine, 3-[(3-cholamidopropyl)-dimethylammonio]-1-propanesulfonate (CHAPS), citrate buffer, dimethyl sulfoxide, 2,2–diphenyl–1–picrylhydrazyl (DPPH), Dulbecco’s phosphate buffered saline (DPBS), ethanol, ethyl acetate, fluorescein di-β-D-galactopyranoside (FDG), gallic acid, glycerol, hexane, hydrochloric acid (HCl), indoxyl acetate, kojic acid, methanol, phosphate buffer, polyethylene glycol (PEG) 8000, D-saccharolactone, tetracycline, thiazol blue tetrazolium bromide (MTT), and tris(hydroxymethyl)aminomethane hydrochloride buffer (TRIS) were obtained from Carl Roth (Karlsruhe, Germany); acarbose, acetylcholinesterase (AChE) from *Electrophorus electricus*; α-amylase from hog pancreas; butyrylcholinesterase (BChE) from equine serum; β-glucuronidase from *Escherichia coli*; and Gram’s iodine solution, lysogeny broth powder (containing 5 mg/mL sodium chloride), rivastigmine, testosterone, and tyrosinase from mushrooms were delivered by Sigma-Aldrich (Steinheim, Germany). 5-Bromo-4-chloro-3-indonyl-β-D-glucuronide was purchased from Carbosynth (Compton-Berkshire, UK). (2S)-2-Amino-3-(3,4-dihydroxyphenyl) propionic acid (levodopa) was obtained from Santa Cruz Biotechnology (Dallas, TX, USA). 17β-Estradiol was obtained from Dr. Ehrenstorfer (Augsburg, Germany). 4-Nitroquinoline-1-oxide was purchased from TCI (Eschborn, Germany). *Aliivibrio fischeri* bacteria (NRRI–B11177, strain 7151) and *Bacillus subtilis* bacteria (DSM-618) were purchased from the German Collection of Microorganisms and Cell Cultures (Leibniz Institute DSMZ, Berlin, Germany). *Salmonella typhimurium* strain TA1535, genetically modified to contain the plasmid pSK1002, was purchased from Trinova Biochem (Giessen, Germany). *Saccharomyces cerevisiae* strain BJ1991 containing the human androgen receptor was obtained from Xenometrix (Allschwil, Switzerland). *Saccharomyces cerevisiae* cells equipped with hERβ were obtained from Erwin Herberle-Bors, University of Vienna, Austria. Additional chemicals and reagents used for pYAS/pYES cell culture have been reported previously [40,42,48]. All the chemicals are of analytical grade, and all the solvents are of chromatographic grade. Bidistilled water was prepared using a Heraeus Destamat Bi-18 E (Thermo Fisher Scientific, Dreieich, Germany). HPTLC plates silica gel 60 F_254_ (20 cm × 10 cm) and HPTLC plates silica gel 60 (20 cm × 10 cm) were provided by Merck (Darmstadt, Germany). The 15 commercially available golden root samples were purchased in Poland and Germany from different vendors (Table 1).

### 3.2. Sample Preparation

The samples (Table 1) were ground (8000 rpm, 5 min, Tube Mill, IKA, Staufen, Germany) and stored in a dark, well-ventilated place at room temperature. Each sample (500 mg) was extracted with 5 mL methanol–water 4:1 (*v*/*v*) in a conical 15-mL Eppendorf tube, vortexed for 1 min, ultrasonicated for 15 min (20 °C, 100%, 480 W, 35 kHz, Sonorex Digi plus DL 255H, Bandelin, Germany) and centrifuged for 5 min (3000× *g*, Heraeus Labofuge 400, Thermo Scientific, Dreieich, Germany). Each supernatant was stored at −20 °C. 

### 3.3. HPTLC−UV/Vis/FLD−EDA Profiling Method

HPTLC plates were pre-washed (developed) with methanol–water 4:1 (*v*/*v*) up to the upper plate edge (Simultan Separating Chamber, biostep, Burkhardtsdorf, Germany) and dried in an oven at 110 °C for 20 min and subsequently wrapped in aluminum foil and stored in a desiccator. The samples (0.2–6.0 µL, as mentioned) were applied as 8 mm bands or, for triplex assays, 12 mm bands (dosage speed 200 nL/s, distance from the lower edge 10 mm, from side edge 16 mm, and between tracks 12 mm, or 22 mm for triplex assays, ATS 4, CAMAG, Muttenz, Switzerland) on HPTLC plates silica gel 60 with or without F_254_. After the samples were dried with a hairdryer for 3 min, development was carried out with ethyl acetate–methanol–water–acetic acid 70:15:15:1 (*v*/*v*/*v*/*v*) up to 70 mm migration distance (Twin Trough Chamber 20 cm × 10 cm, CAMAG). After separation, the chromatogram was dried for 10 min (ADC 2, CAMAG). Then the chromatogram was documented (TLC Visualizer, CAMAG) at 254 nm (UV), 366 nm (FLD), and under white light illumination (VIS).

Eleven chromatograms were prepared analogously with a few adjustments depending on the assay, as mentioned. Each chromatogram was neutralized with 5% sodium bicarbonate pH 8) or sodium acetate buffer (pH 7) by means of piezoelectric spraying (2.5 mL, yellow nozzle, level 6, Derivatizer, CAMAG) followed by drying for 3 min (hairdryer) and 20 min (ADC 2, CAMAG). The neutralized chromatograms were sprayed with the respective assay solutions and incubated in a humid atmosphere (KIS polypropylene box, 27 cm × 16 cm × 10 cm, ABM, Wolframs–Eschenbach, Germany). For each assay, a respective positive control was applied [40,41]. Each assay was performed at least twice to confirm the reproducibility of the response.

The *A. fischeri* bioassay was performed according to [41,49]. After spraying the *A. fischeri* suspension (4 mL, red nozzle, level 6), the still-humid plate was transferred to the BioLuminizer (CAMAG). Ten images were recorded over 30 min (exposure time 60 s, trigger interval 3.0 min). Caffeine was used as a positive control (1 mg/mL in methanol; 0.5, 1.5, and 3 μL/band).

The *B. subtilis* bioassay was performed as previously described [40]. The bacteria suspension (100 µL cryostock in 20 mL 2.3% Müller–Hinton broth incubated overnight at 37 °C and adjusted to optical density 1.1 at 600 nm) was sprayed (3 mL, red nozzle, level 6), and this was followed by incubation at 37 °C for 2 h. The MTT substrate solution (0.2% in DPBS buffer) was sprayed (0.5 mL, blue nozzle, 6 level), and this was followed by incubation at 37 °C for 30 min, plate drying (50 °C, 10 min, TLC Plate Heater, CAMAG), and documentation at white light illumination. Tetracycline was used as a positive control (0.005 mg/mL in ethanol; 0.5, 1.5 and 3 µL/band).

The planar SOS-Umu-C genotoxicity bioassay was performed on HPTLC plates without a fluorescence indicator as previously described [50,51,52]. The *Salmonella* suspension was sprayed (2.5 mL, yellow nozzle, level 3) on the plate, and this was followed by incubation at 37 °C for 3 h. The FDG substrate solution (25 µL of 0.5% FDG in dimethyl sulfoxide in 2.5 mL phosphate buffer) was sprayed (2.5 mL, red nozzle, level 6), and this was followed by incubation at 37 °C for 15 min, plate drying and documentation at 254 nm. 4-Nitroquinoline-1-oxide was used as a positive control (1 ng/mL in methanol; 10 µL/band).

The tyrosinase inhibition assay was performed as previously described [53]. The substrate solution (4.5 mg/mL levodopa in 20 mM phosphate buffer pH 6.8 plus 2.5 mg of CHAPS and 7.5 mg PEG 8000) was sprayed (2 mL, blue nozzle, level 5). After plate drying (2 min), the tyrosinase solution (400 U/mL in phosphate buffer) was sprayed (2 mL, blue nozzle, level 5), and this was followed by incubation at room temperature in the dark for 20 min. Kojic acid was used as a positive control (0.1 mg/mL in ethanol; 1, 3, and 6 μL/band).

The AChE/BChE inhibition assays were performed as previously described [54]. The substrate solution (1 mg/mL indoxyl acetate in ethanol) was sprayed (2 mL, green nozzle, level 6), and this was followed by drying for 3 min and then spraying with 3 mL enzyme solution (6.66 U/mL AChE or 3.34 U/mL BChE, each in Tris–HCl buffer plus 1 mg/mL bovine serum albumin). The incubation at 37 °C took 1 h. After drying (10 min in ADC 2), the plate was documented at white light illumination. Rivastigmine was used as a positive control (0.1 mg/mL in methanol; 2, 4, and 8 μL/band).

The β-glucuronidase inhibition assay was performed as previously described [40]. The enzyme solution (50 U/mL in 0.1 M potassium phosphate buffer pH 7 plus 1 mg/mL bovine serum albumin) was sprayed (2 mL, yellow nozzle, level 6), and this was followed by incubation at 37 °C for 15 min, spraying with 1.5 mL substrate solution (2 mg/mL 5-bromo-4-chloro-3-indonyl-β-D-glucuronide in water), incubation at 37 °C for 1 h, plate drying (10 min in ADC 2), and documentation at white light illumination. D-saccharolactone solution was used as a positive control (0.1 mg/mL in water; 0.8, 1.5, and 3 µL/band).

The DPPH• assay was performed by spraying the chromatogram with DPPH• solution (4 mL, 0.04% in methanol, green nozzle, 4 level) and then drying (10 min in ADC 2) and documenting it at white light illumination. Gallic acid was used as a positive control (0.25 mg/mL in methanol; 0.2, 0.6, and 1.0 µL/band).

The α-amylase inhibition assay was performed as recently described [40]. The enzyme solution (62.5 U/mL in sodium acetate buffer, pH 7) was sprayed (2 mL, red nozzle, level 6), and this was followed by incubation at 37 °C for 30 min. Then, the substrate solution (2% starch in water) was sprayed (1 mL, red nozzle, level 6), and this was followed by another incubation at 37 °C for 20 min and by spraying with Gram’s iodine solution (0.5 mL, yellow nozzle, level 6). Acarbose was used as a positive control (0.1 mg/mL in methanol; 0.3, 0.6, and 0.9 μL/band).

The triplex pYAVES/pYAVAS bioassays were performed on HPTLC plates without a fluorescence indicator, according to [42]. The samples were applied as a 12 mm band (6 μL/band, track distance 22 mm) and developed with ethyl acetate–methanol–water 70:15:15 (*v*/*v*/*v*). After plate drying, two stripes (1 mm × 70 mm) were sprayed along each separate sample track (Freemode option, winCATS software). The first agonist stripe (considered also as a positive control) was 17β-estradiol (5 μL, 10 ng/mL in ethanol) for the pYAVES, while testosterone was used (4 μL, 5 μg/mL in methanol) for the pYAVAS. The second end-product stripe was fluorescein (2 μL, 50 μg/mL in methanol), which was used to detect false-positive responses. The dried chromatogram was immersed in a fixation solution (0.25% Degalan in *n*-hexane) for 10 min, dried for 10 min, sprayed with 2.5 mL Tween 20 solution (0.02% in ethanol), and dried for 10 min. The yeast cell suspension was sprayed (2.8 mL, red nozzle, level 6) on the plate, incubated at 30 °C for 3 h (pYAVES) or 4 h (pYAVAS), sprayed with FDG solution (2.5 mL, yellow nozzle, level 6), incubated at 37 °C for 15 min, and dried for 10 min. The resulting bioautogram was documented at FLD 254 nm.

### 3.4. HPTLC−HPLC−HESI-HRMS

Golden root extract IDs 1 and 6 (4 µL/band) were applied in triplicate and separated as described for the respective assay. The zones marked were eluted with 10% methanol in an aqueous solution at a flow rate of 0.1 mL/min for 1 min (open-source modified autoTLC-MS Interface [43]). Subsequently, the analytes were transferred through a 50 µL sample loop and Accucore RP-MS, 10 mm × 2.1 mm, 2.6 μm (Thermo Scientific, Bellefonte, PA, USA) desalting cartridge to the HPLC separation [40,42]. An Accucore RP-MS (100 mm × 2.1 mm, 2.6 μm, Thermo Scientific, Bellefonte, PA, USA) analytical column was used. Eluent phase A (2.5 mM ammonium acetate in water, pH 4.5) and eluent phase B (methanol) were used for gradient elution over 12 min using the following program: 0–2 min 2% B, 2–7 min increase from 2% to 100% B; 10–12 min from 100 to 2% B to restore initial gradient composition. The flow rate was 0.4 mL/min, and the column temperature was set to 40 °C. Full scan mass spectra were in the positive and negative ionization modes at mass range *m*/*z* 100–1100 recorded using the HESI-HRMS system (Q Exactive Plus mass spectrometer, Thermo Fisher Scientific, Bellefonte, PA, USA). The following parameters were used: capillary temperature 270 °C, spray voltage ± 3.5 kV, sheath gas 20 arbitrary units, and aux gas 10 arbitrary units (S–Lens RF level 50). The spectra were evaluated using Xcalibur 3.0.63 software (Thermo Fisher Scientific, Bellefonte, PA, USA).

## 4. Conclusions

The differences and variances in the 15 characteristic bioactivity profiles of 15 different golden root products obtained on the market highlighted the importance of effect-directed profiling as a quality control measure for plant-based supplements. Interestingly, the USP reference product showed medium activity in most assays. The activities of three samples (IDs 13–15) were comparably poor, except in respect of α-amylase inhibition. This was partially explained by the lower amount of dry root extract or root powder contained in the products, whereby root powder is comparatively less active than root extract. In particular, the genotoxicity bioassay pointed to a genotoxic zone in two products (IDs 1 and 7) tentatively assigned as hydroquinone. These results point to the need for bioactivity profiling of supplements before they are made available to consumers. Antioxidants and antibacterials against *B. subtilis* and *A. fischeri* were detected, in addition to inhibitors of acetylcholinesterase, butyrylcholinesterase, β-glucuronidase, α-amylase, and tyrosinase. Information was obtained for the first time about β-glucuronidase, α-amylase and butyrylcholinesterase inhibitors, and genotoxic compounds, in addition to individual estrogens and antiestrogens in golden root products. Even in 600 µg of the golden root extract, no androgenic or antiandrogenic activity was observed. The main bioactive compounds of the golden root detected and tentatively assigned were salidroside, viridoside, rosavin, rosarin, rosin, rosiridin, rhodioloside D, stearic acid, and palmitic acid.

## Figures and Tables

**Table 1:** The list of the 15 commercially available golden root samples studied and respective information provided on the label.

ID	Sample Form	Dosage Form	Product Name	Manufacturer, City, Country	Plant Origin	Batch Number	Expiration Date
1	Root powder	Powder	Rhodiola-Rosenwurz	Vitaideal Vegan, Enshede, Netherlands	Netherlands	-	-
2	Root dry extract 25:1	Capsule	Rhodiola rosea	Green Naturals, Berlin, Germany	Germany	-	-
3	Root dry extract 20:1	Powder	Różeniec górski	Proherbis, Zarzecze, Poland	China	-	04/2023
4	Dried root	Dried root	Korzeń różeńca górskiego	Farmvit, Peterborough, UK	-	-	-
5	Root dry extract, 520 mg	Capsule	Różeniec górski	Medica herbs, Cracow, Poland	-	ROZ-0-01	02/2024
6	Root dry extract, 500 mg	Capsule	Rhodiola	Now Foods, Chicago, USA	-	3196374	02/2026
7	Dried rhizome	Dried rhizome	Kłącze różeńca górskiego	Dary natury, Grodzisk, Poland	-	-	06/2023
8	Root and rhizome dry extract	Powder	Rhodiola rosea root and rhizome dry extract	Sigma Aldrich	Canada	F00100	-
9	Dried root and rhizome	Dried root/ rhizome	Różeniec górski	Natvita, Mirków, Poland	Russia	29995	02/2023
10	Powdered root, 400 mg	Capsule	Rhodiola	Fushi, London, UK	China	FOHC6180/12970CN	09/2023
11	Root dry extract, 250 mg, with additives	Capsule	Rhodiola	Solgar Leonia, USA	-	530923-02	12/2023
12	Root dry extract, 500 mg, with inulin	Capsule	Rhodiola	ForMeds, Poznań, Poland	-	K220221	02/2023
13	Root dry extract, 140 mg, with additives	Capsule	Rhodiola	Pharmovit, Płock, Poland	-	RG0719/ PH	07/2023
14	Root dry extract, 100 mg	Tablet	Różeniec górski	Herbapol, Lublin, Poland	-	010621	06/2023
15	Root powder, 225 mg	Tablet	Arktyczny korzeń	Altermedica Laboratories, Żywiec, Poland	-	A0419/7	05/2023

**Table 2:** HPTLC−HPLC−HESI-HRMS signals obtained in the positive and negative ionization modes and the tentative assignment of the active compound zones **I**−**VI** in the golden root product IDs 1 and 6 (400 µg/band each).

Zone ID	*hR_F_*	Bioactivity	Tentative Assignment	Formula	Calculated Mass [Da]	Observed Mass [Da]	Mass Error (Δ ppm)	Adduct Ions
I 6	20	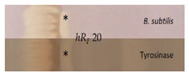	Rhodioloside D	C_16_H_30_O_8_	350.1941	409.2084 373.1828	−1.09 1.13	[M+CH_3_COO]^−^ [M+Na]^+^
II 6	35	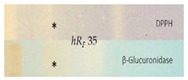	Rosavin/rosarin	C_20_H_28_O_10_	428.1683	427.1610 451.1576	−0.01 −0.26	[M-H]^−^ [M+Na]^+^
III 6	51	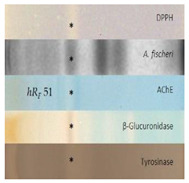	Rosiridin	C_16_H_28_O_7_	332.1835	391.1978 355.1722	−1.33 1.54	[M+CH_3_COO]^−^ [M+Na]^+^
			Viridoside	C_15_H_22_O_7_	314.1365	359.1351 337.1258	−1.06 −0.10	[M+CHOO]^−^ [M+Na]^+^
			Salidroside	C_14_H_20_O_7_	300.1209	299.1139 323.1096	−0.74 1.57	[M-H]^−^ [M+Na]^+^
IV 6	61	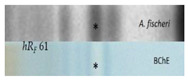	Rosin	C_15_H_20_O_6_	296.1260	355.1399 319.1146	−0.02 1.98	[M+CH_3_COO]^−^ [M+Na]^+^
V 1	93	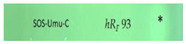	Hydroquinone	C_6_H_6_O_2_	110.0368	109.0294 111.0444	0.68 −2.74	[M-H]^−^ [M+H]^+^
VI 6	23	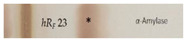	Stearic acid	C_18_H_36_O_2_	284.2715	283.2643	−0.13	[M-H]^−^
			Palmitic acid	C_16_H_32_O_2_	256.2402	255.2330	−0.26	[M-H]^−^

## Data Availability

Data are available upon reasonable request.

## Supplementary Materials

The following supporting information can be downloaded at: https://www.mdpi.com/article/10.3390/molecules28041535/s1, Figure S1: HPTLC–Vis chromatogram via Fast blue B salt reagent; Figure S2: DPPH•–Vis autogram; Figure S3: HPTLC–FLD–α–/β–glucosidase inhibition autograms; Figure S4: HPTLC–Vis–α-amylase inhibition autogram; Figure S5: HPTLC−HPLC−HESI-HRMS spectra; Table S1: Mobile phase optimization.

## Sample Availability

Samples are available from the authors on request.
