# Supplementary material for: Bioactivity Profiles on 15 Different Effect Mechanisms for 15 Golden Root Products via High-Performance Thin-Layer Chromatography, Planar Assays, and High-Resolution Mass Spectrometry
Source: Molecules. 2023 Feb 5;28(4):1535. doi: 10.3390/molecules28041535 (PMC9959789; doi:10.3390/molecules28041535)
Supplement: Supplementary file 1 [file molecules-28-01535-s001.zip › molecules-2108198-supplementary.pdf]

Supplementary Material

Bioactivity Profiles on 15 Different Effect Mechanisms for 15 Golden Root Products via High-Performance Thin-Layer Chromatography, Planar Assays, and High-Resolution Mass Spectrometry

Hanna Nikolaichuk [1-3], Irena M. Choma [2] and Gertrud E. Morlock [1,*]

[1] Chair of Food Science, Institute of Nutritional Science, Justus Liebig University Giessen,

Heinrich-Buff-Ring 26-32, 35392 Giessen, Germany

[2] Department of Chromatography, Faculty of Chemistry, Maria Curie-Sklodowska University

Maria Curie-Sklodowska sq.3, 20031 Lublin, Poland

[3] Department of Bioanalytics, Faculty of Biomedicine, Medical University of Lublin,

Jaczewskiego st. 8b, 20090, Lublin, Poland

***** Correspondence: Gertrud.Morlock@ernaehrung.uni-giessen.de

Table S1 Mobile phase optimization for a golden root extract (5 µL/band or 3−4 µL/band) separated on the HPTLC plate silica gel 60 F_{254} with the specified mobile phase system

MP	Solvent composition	Ratio (*V/V*)	Chromatogram at 254 nm, FLD 366 nm and bioluminescence after *Aliivibrio fischeri* bioassay
1	Ethyl acetate-methanol-water	7.7:1.3:1	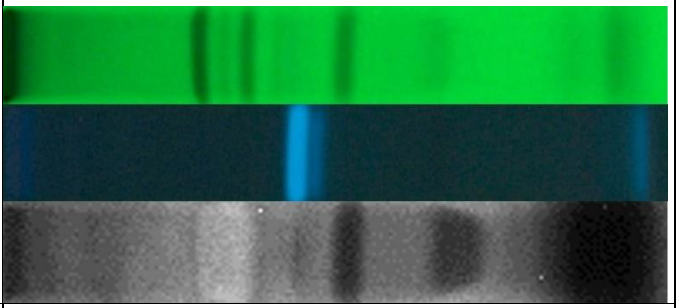
2	Ethyl acetate-methanol-water-acetic acid	7.7:1.3:1:0.1	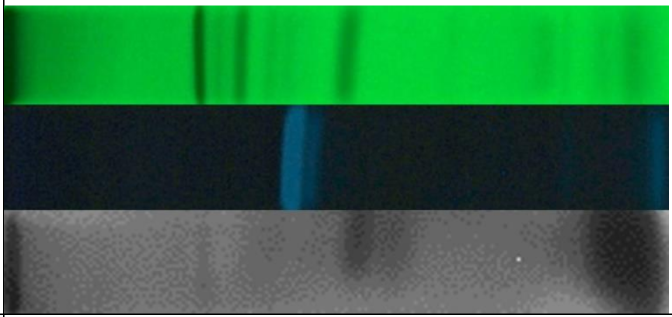
3	Ethyl acetate-methanol-water-acetic acid	7.7:1.3:1:0.2	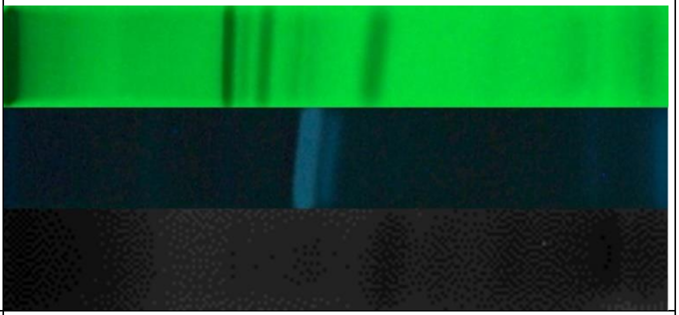
4	Ethyl acetate-acetonitrile-water	7:2:1	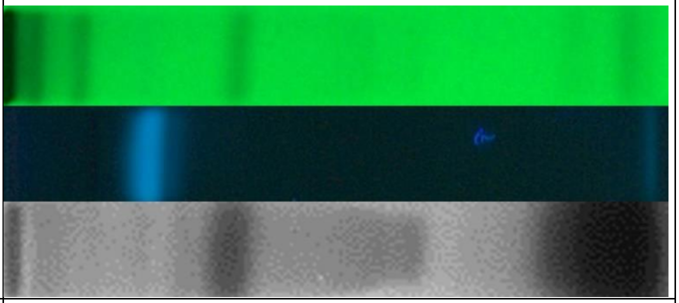
5	Ethyl acetate-acetonitrile-water	5:4:1	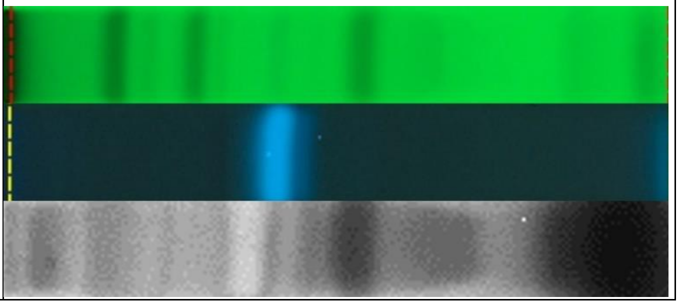

6	Ethyl acetate-methanol-water-acetic acid	7.7:1.3:1:0.15	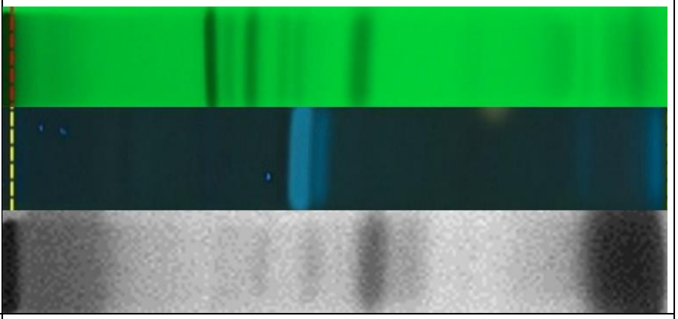
7	Ethyl acetate-methanol-water-acetic acid	7:1.5:1:0.2	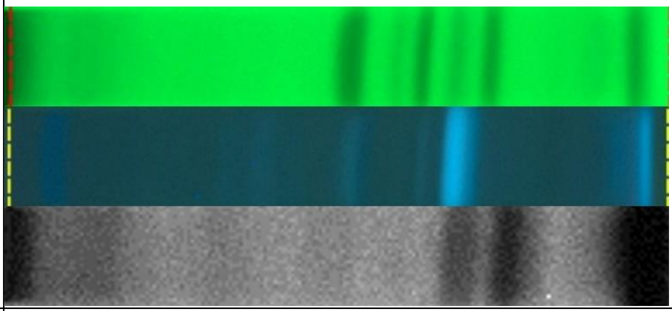
8	Ethyl acetate-acetonitrile-water-acetic acid	5:4:1:0.1	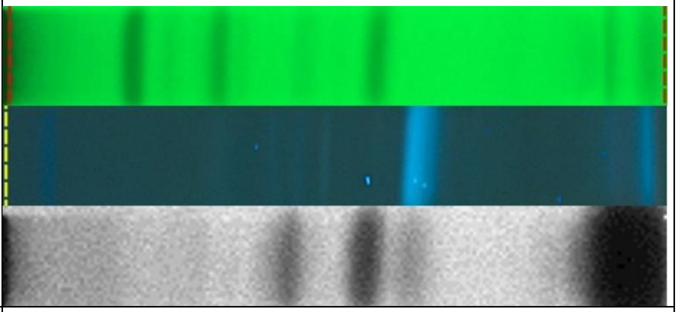
9	Ethyl acetate-methanol-water-acetic acid	7:1.5:1:0.1	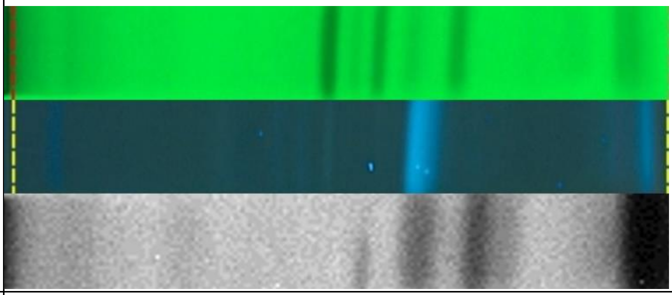
10	Ethyl acetate-methanol-water-acetic acid	5:4:1:0.1	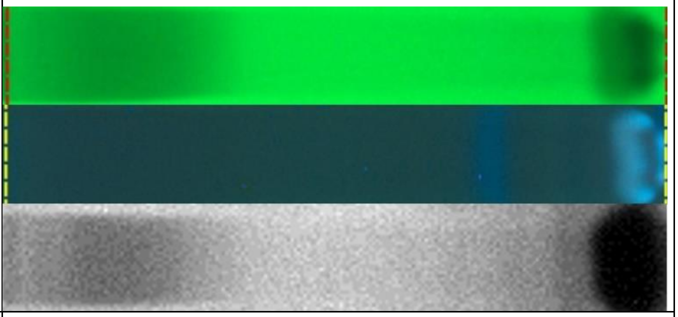
11	Ethyl acetate-methanol-water-acetic acid	6:3:1:0.1	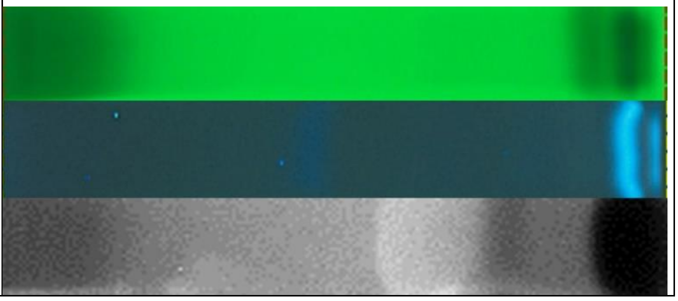

12	Ethyl acetate-methanol-water-acetic acid	7:2:1:0.1	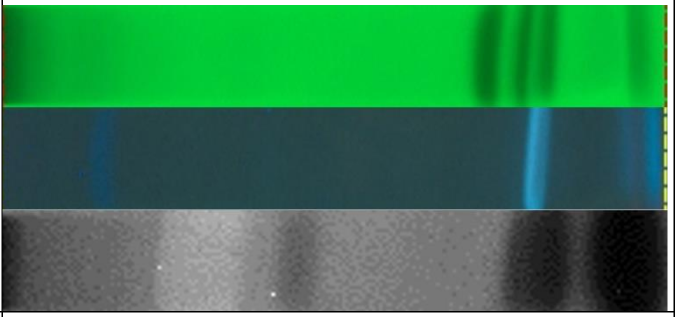
13	Ethyl acetate-acetonitrile-water-acetic acid	4:5:1:0.1	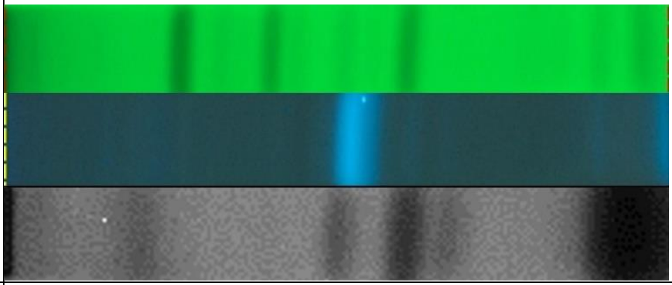
14	Focusing (methanol), 3 cm<br><br>Ethyl acetate-water	 <br>10:1	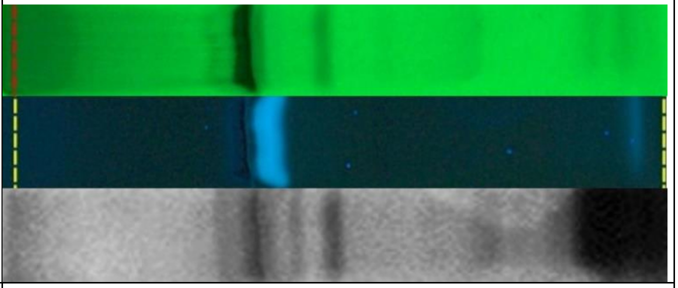
15	Focusing (acetone, 2 x methanol), 2 cm<br><br>Ethyl acetate-methanol-water-acetic acid	 <br>7:1.5:1.5:0.1	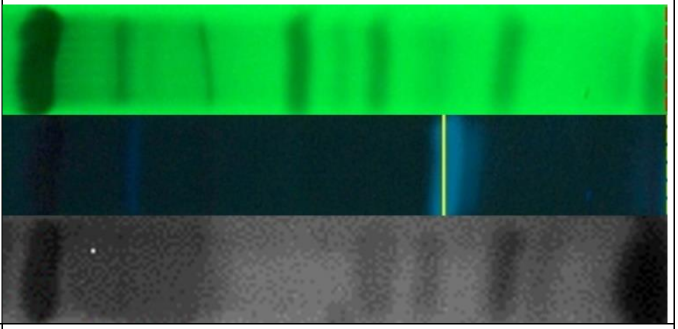
16	Ethyl acetate-methanol-water-acetic acid	7:1.5:1.5:0.1	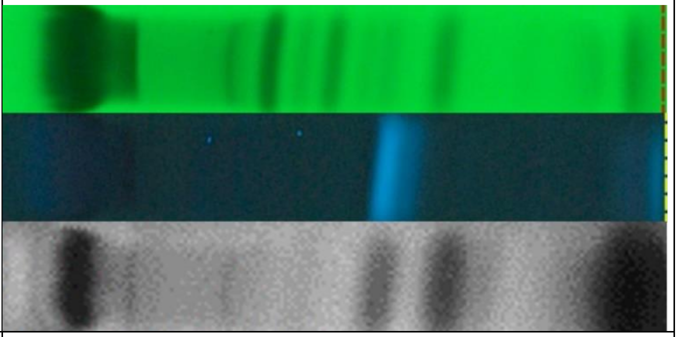
17	Focusing (2 x acetone, methanol), 2 cm<br><br>Ethyl acetate-methanol-water-acetic acid	 <br>7:1.5:1.5:0.1	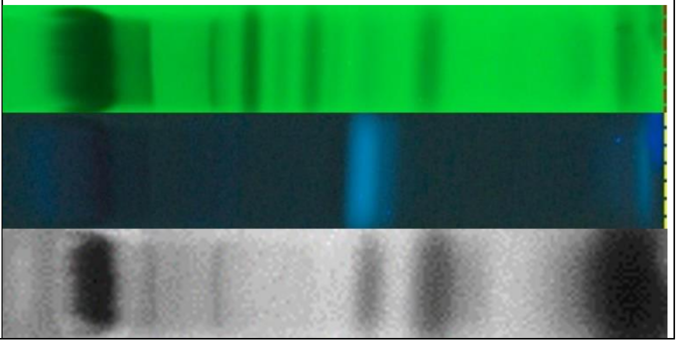

18	Ethyl acetate-methanol-water-acetic acid	7:1.5:1.5:0.1	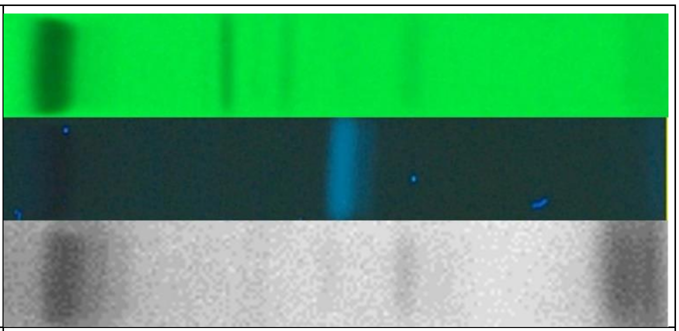
19	Focusing (2 x acetone, methanol), 2 cm<br><br>Ethyl acetate-methanol-water-acetic acid	 <br>7:1.5:1.5:0.1	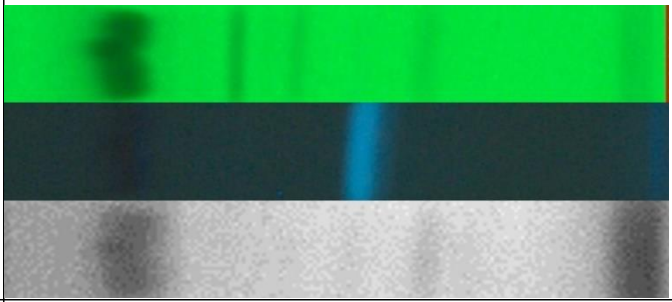
20	Ethyl acetate-methanol-water-acetic acid	7:1.5:1.5:0.1	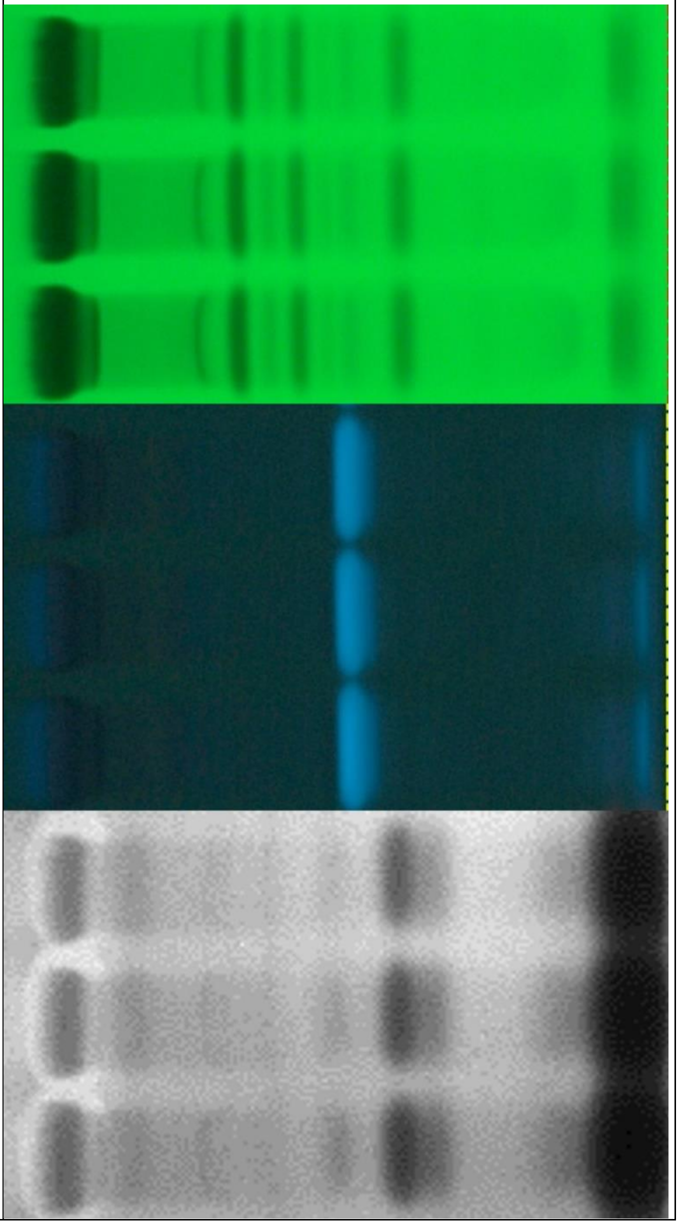

HPTLC–Vis nm chromatogram sprayed with *p*–anisaldehyde sulphuric acid (for comparison)

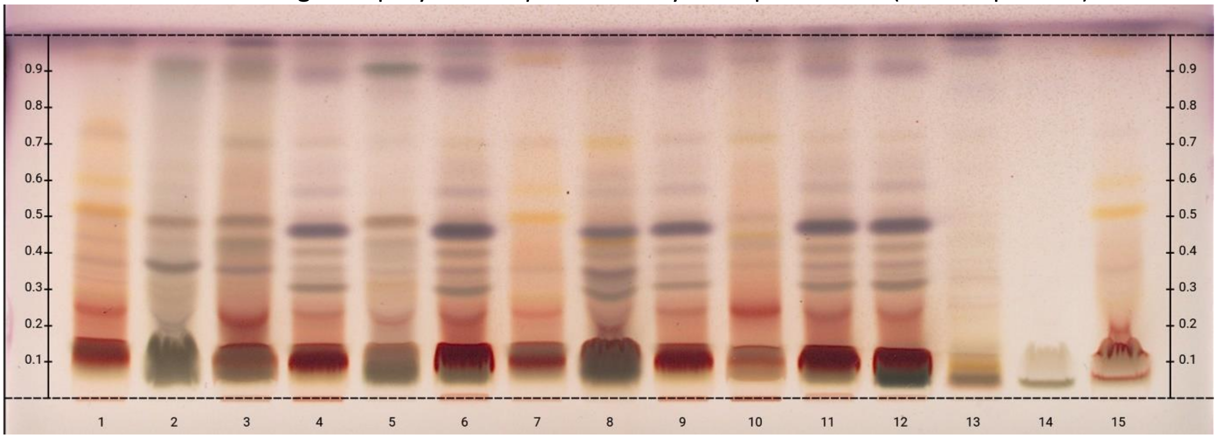

HPTLC–FLD 366 nm chromatogram sprayed with *p*–anisaldehyde sulphuric acid (for comparison)

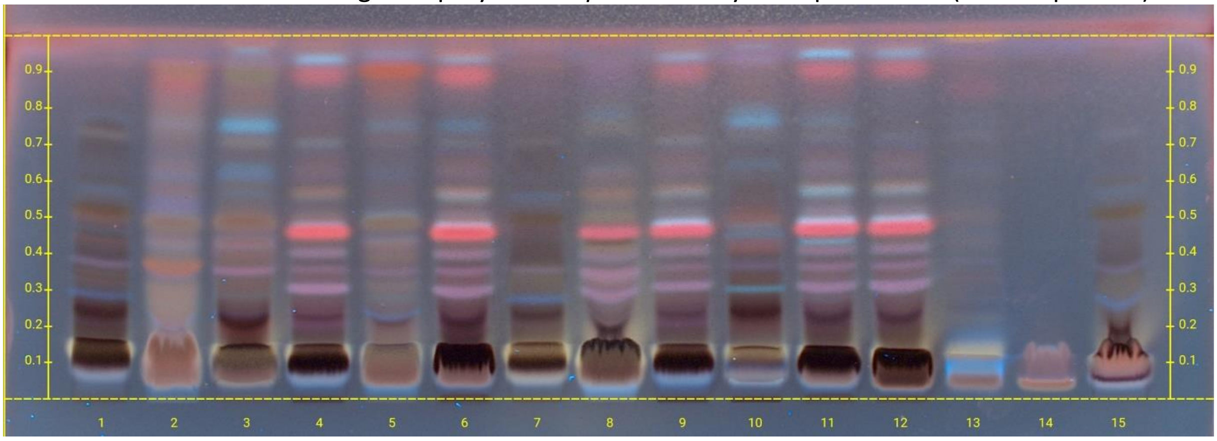

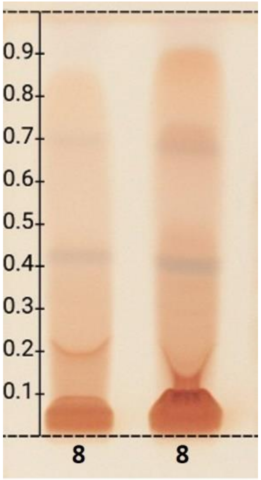

Figure S1. HPTLC–Vis chromatogram after derivatization via Fast Blue B salt reagent (0.5% aqueous solution) of a golden root extract ID 8 (200 and 400 µg/band) separated on HPTLC plate silica gel 60 F_{254} with ethyl acetate-methanol-water-acetic acid 70:15:15:1 (*V/V/V/V*) [GM1]and detected at white light illumination.

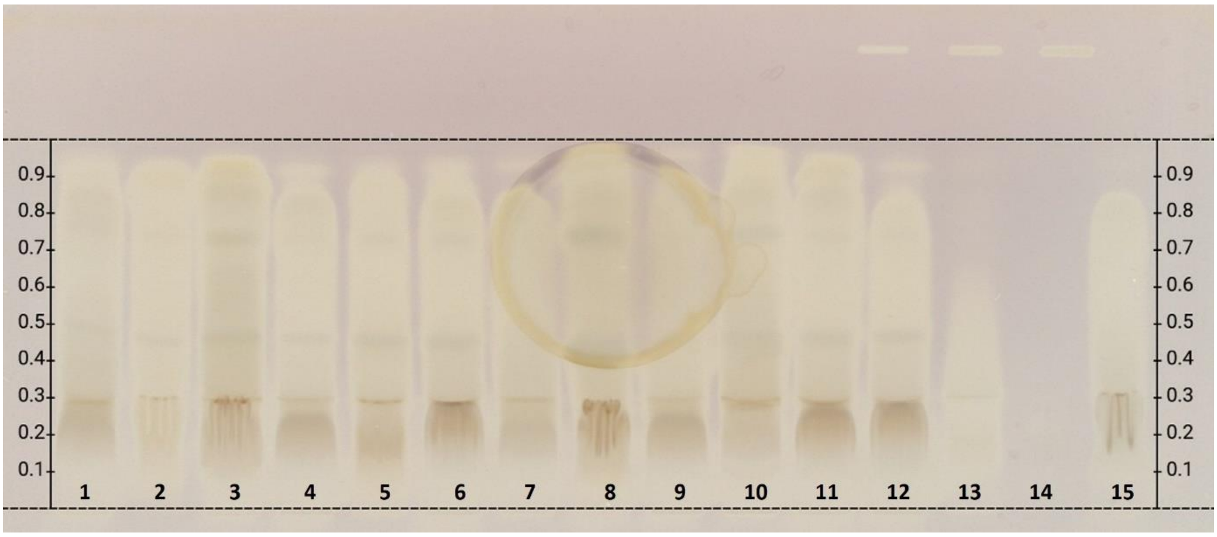

Figure S2. DPPH•–Vis autogram of golden root extract IDs 1–15 (400 µg/band each) separated on HPTLC plate silica gel 60 F_{254} with ethyl acetate-methanol-water-acetic acid 70:15:15:1 (*V/V/V/V*) and detected at white light illumination after the DPPH• assay. Positive control gallic acid in the right upper plate corner. The ring in the plate middle was caused by misfunction of the Derivatizer nozzle.

HPTLC–Vis nm chromatogram (for comparison)

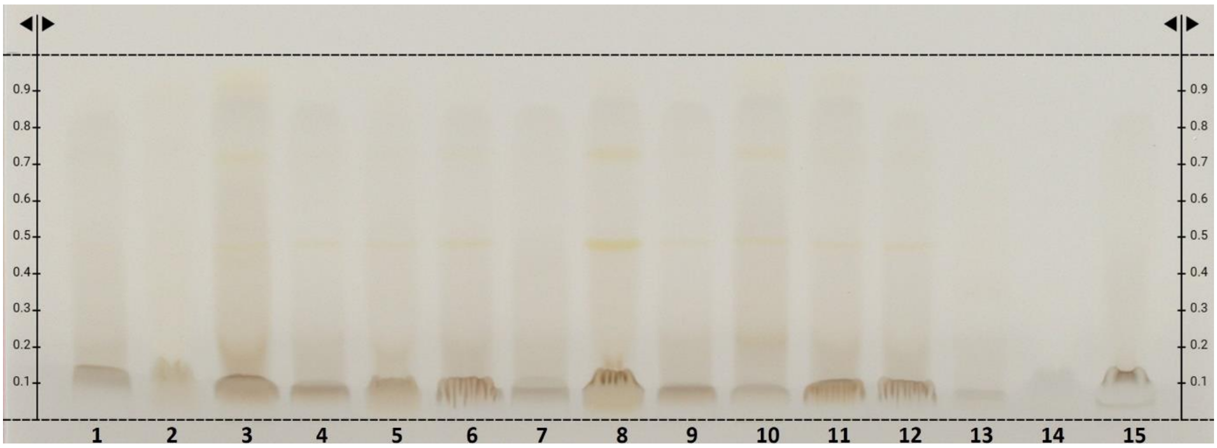

HPTLC–UV 254 nm chromatogram (for comparison)

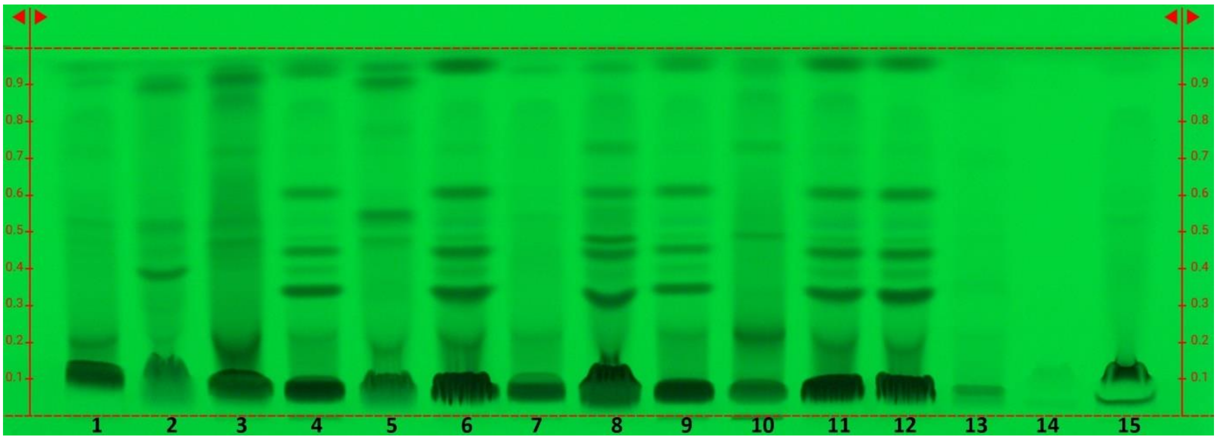

HPTLC–FLD 366 nm chromatogram (for comparison)

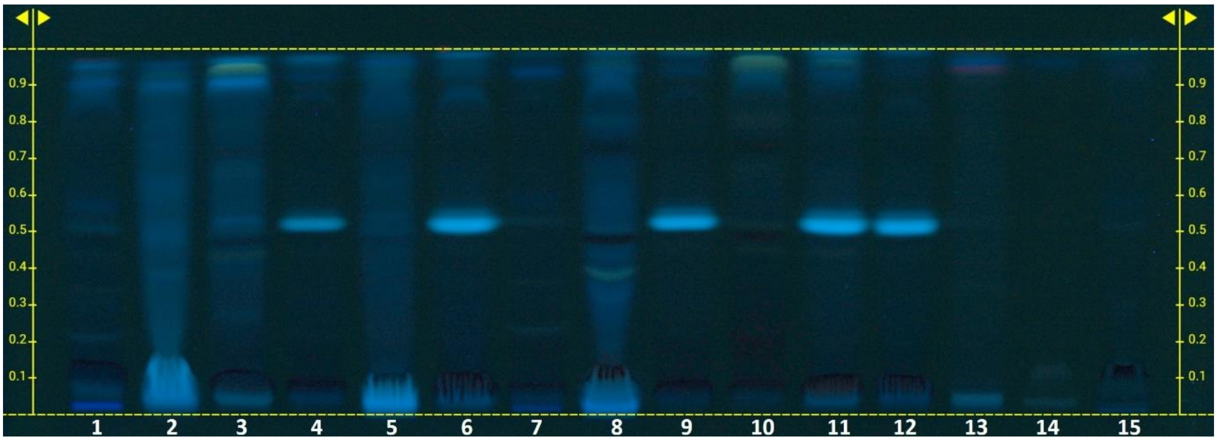

Figure S3. HPTLC–α/β-glucosidase inhibition autograms at FLD 366 nm in comparison to HPTLC–Vis/UV/FLD chromatograms of golden root extracts IDs 1–15 (400 µg/band each) separated on HPTLC plate silica gel 60 F_{254} with ethyl acetate-methanol-water-acetic acid 70:15:15:1 (*V/V/V/V*) and detected at FLD 366 nm after the respective glucosidase assay *versus* only sprayed with 4-methylumbelliferone solution. The positive controls (applied at plate top as ethanolic solution) were acarbose (3, 9, and 18 µg/band) and imidazole (2, 5, and 8 µg/band) for the α/β–glucosidase, respectively.

HPTLC–α-glucosidase inhibition autogram at FLD 366 nm

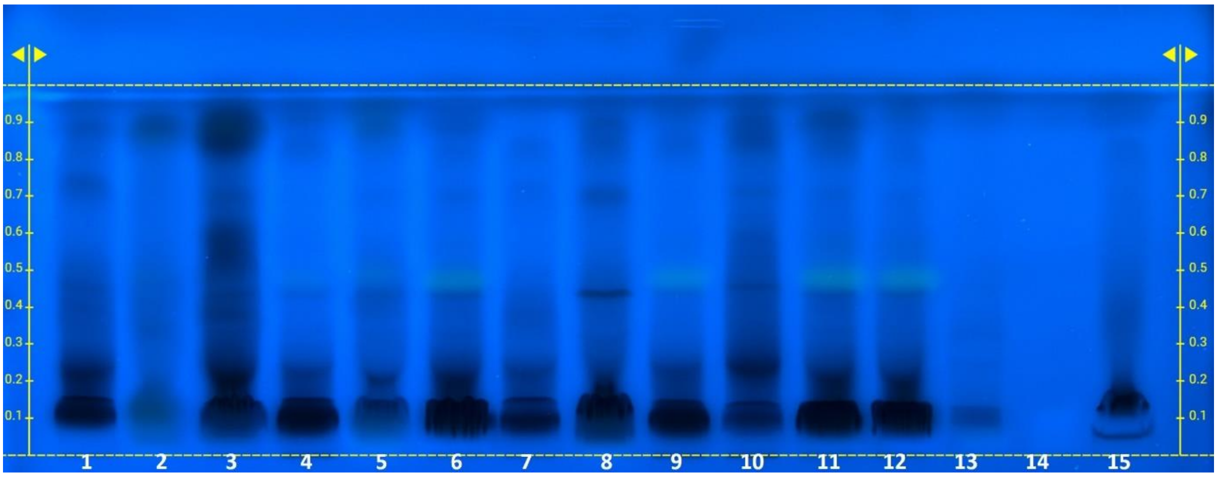

HPTLC–β-glucosidase inhibition autogram at FLD 366 nm

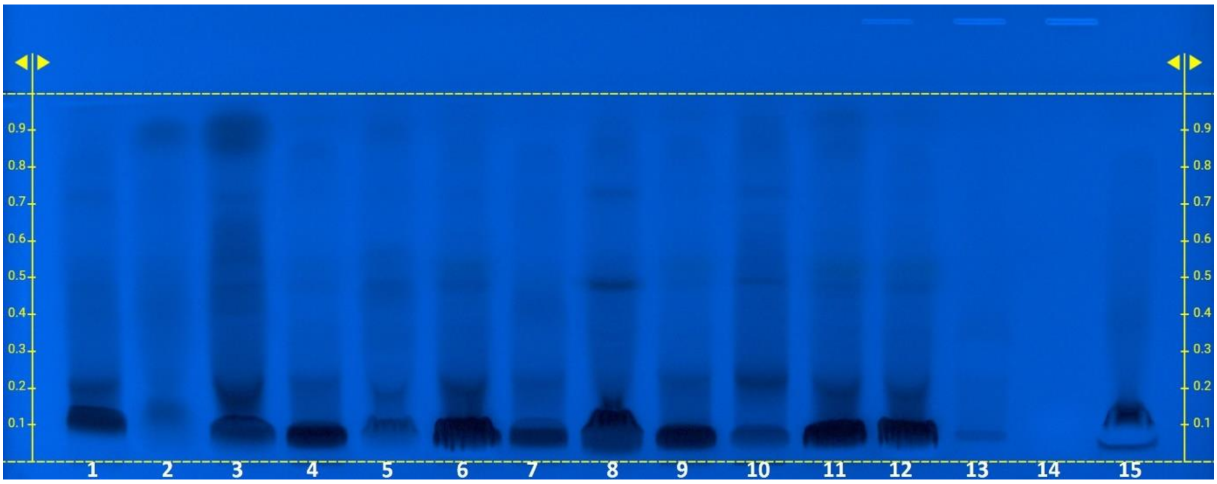

HPTLC–FLD 366 nm chromatogram sprayed with 4-methylumbelliferone solution

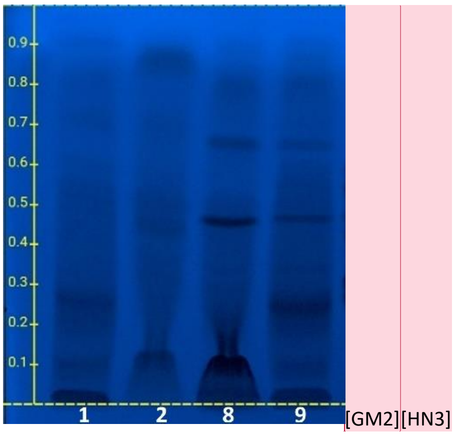

Figure S3. Continued.

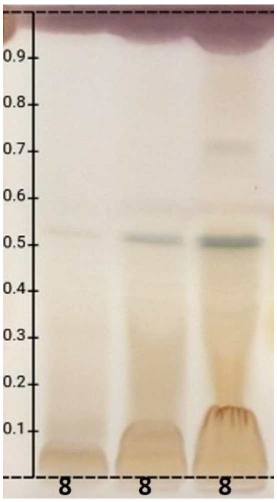

Figure S4. HPTLC–α-amylase inhibition autogram of golden root extract ID 8 (100, 200 and 400 µg/band) separated on HPTLC plate silica gel 60 F_{254} with ethyl acetate-methanol-water-acetic acid (70:15:15:1, *V/V/V/V*) and detected at white light illumination after the α-amylase inhibition assay.

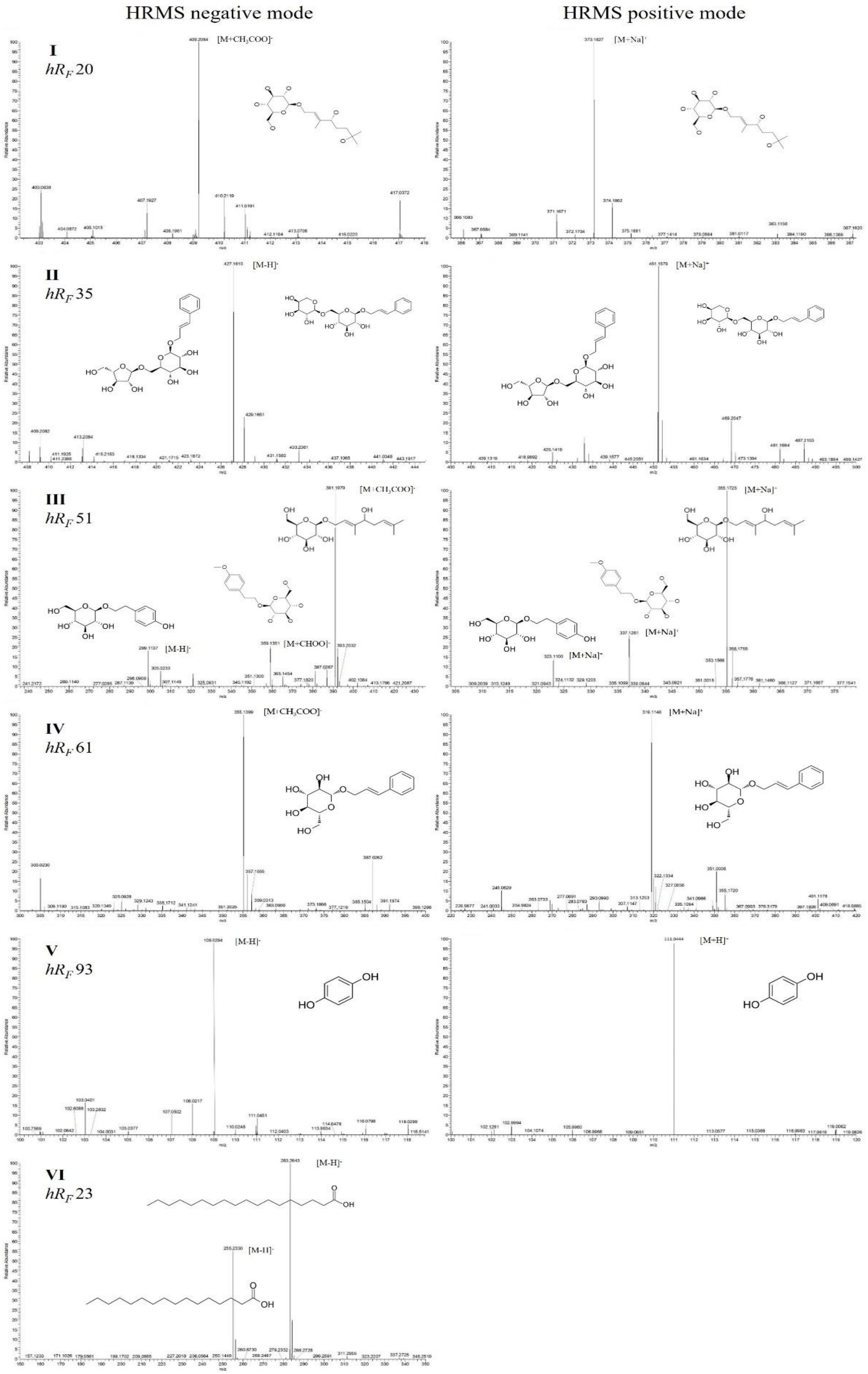

Figure S5. HPTLC−HPLC−HESI-HRMS spectra of selected bioactive zones
